# Association of the cholesterol-high-density lipoprotein-glucose index with coronary atherosclerotic burden in patients with coronary artery disease

**DOI:** 10.3389/fnut.2026.1906236

**Published:** 2026-08-25

**Authors:** Jiayuan Zhang, Hongxin Dong, Shengyi Lei, Yan Lu

**Affiliations:** 1 Department of Cardiology, First Hospital of Shanxi Medical University, Taiyuan, Shanxi, China; 2 School of Computer Science and Technology, North University of China, Taiyuan, Shanxi, China; 3 School of Public Health, Shanxi Medical University, Taiyuan, Shanxi, China

**Keywords:** CHG index, coronary artery disease, coronary atherosclerosis, Gensini score, metabolic marker

## Abstract

**Background:**

The cholesterol-high-density lipoprotein-glucose (CHG) index is a novel composite metabolic marker, but its association with coronary atherosclerotic burden in patients with coronary artery disease (CAD) remains unclear. This study aimed to investigate the association between the CHG index and the Gensini score in patients with CAD.

**Methods:**

We included patients with CAD admitted between January 2024 and January 2025. Multivariable linear regression and restricted cubic spline (RCS) analyses were performed to evaluate the association and dose–response relationship between the CHG index and log-transformed (Gensini score + 1). Subgroup and sensitivity analyses assessed the robustness of the findings. Receiver operating characteristic (ROC) curve analysis was used to evaluate discriminative ability for identifying high coronary atherosclerotic burden. Net reclassification improvement (NRI), integrated discrimination improvement (IDI), and decision curve analysis (DCA) were used to assess the incremental value of the CHG index.

**Results:**

A total of 758 participants were included. In the fully adjusted model, each 1-SD increase in the CHG index was associated with a 0.121-unit increase in log-transformed (Gensini score + 1) (*β* = 0.121, 95% CI, 0.036–0.205, *p* = 0.005). RCS analysis further indicated an approximately linear dose–response relationship. Quartile-based analyses showed an overall positive gradient. The association was directionally consistent across subgroups, with no significant interactions, despite not reaching statistical significance in some strata. Sensitivity analyses yielded consistent results. Among the metabolic markers evaluated, the CHG index had the numerically highest standalone AUC, although its discriminative ability was modest (AUC = 0.603). Adding the CHG index to the basic model slightly increased the AUC from 0.652 to 0.667, without a statistically significant difference (DeLong *p* = 0.168), but significantly improved continuous NRI (0.135, *p* = 0.042) and IDI (0.016, *p* = 0.002). DCA indicated a modest net benefit over a limited range of intermediate threshold probabilities.

**Conclusion:**

A higher CHG index was associated with a higher Gensini score in patients with CAD. As a simple and inexpensive metabolic marker, the CHG index may provide complementary information for assessing coronary atherosclerotic burden. However, its clinical utility requires further evaluation in prospective studies incorporating external validation and longitudinal clinical outcomes.

## Graphical abstract

**Figure:**
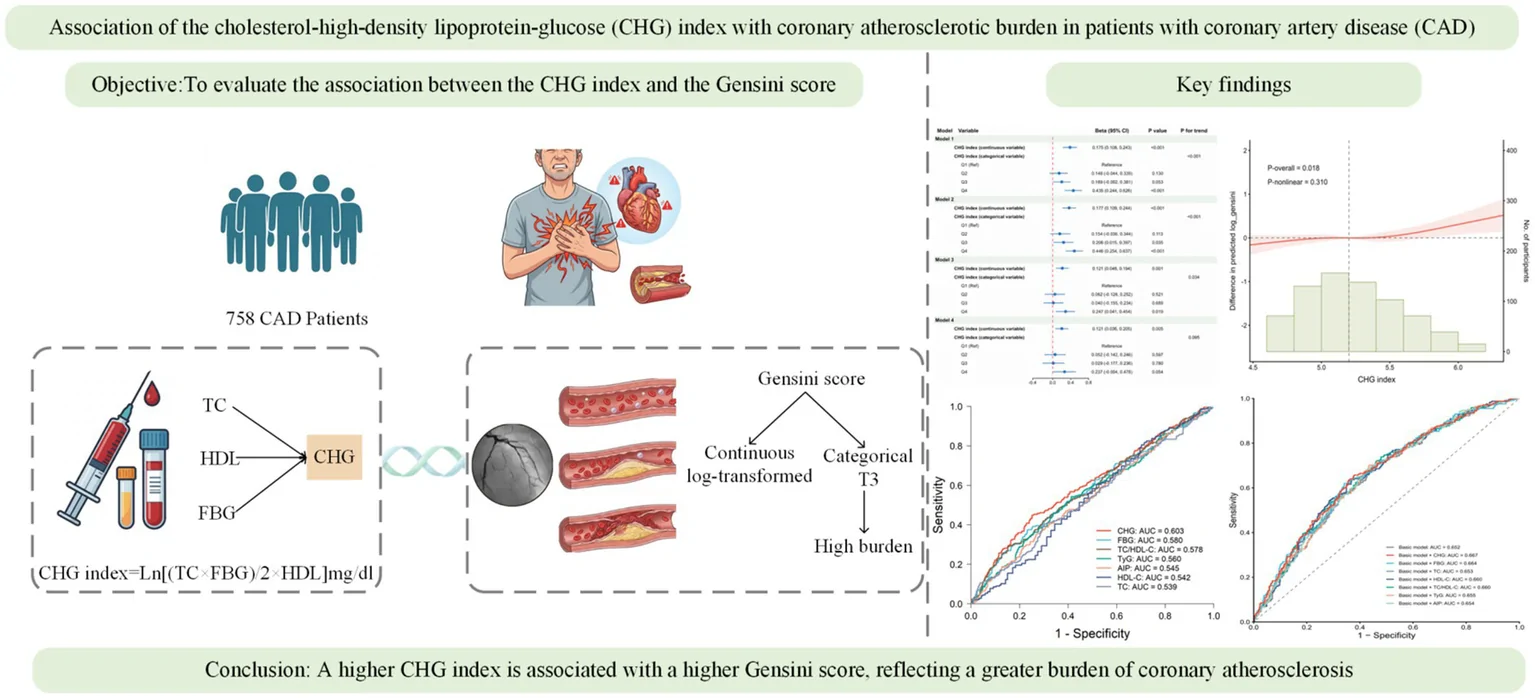

## 1 Introduction

Cardiovascular disease (CVD) remains a major global public health burden. According to the Global Burden of Disease Study, deaths attributable to CVD increased from 12.1 million in 1990 to 18.6 million in 2019 (1). By 2050, the global prevalence of CVD is expected to increase by 90%, while the crude mortality rate is projected to rise by 73.4% (2). Ischemic heart disease and stroke account for most of the disease burden. Coronary artery disease (CAD) is one of the leading causes of mortality and disability worldwide (3). Despite substantial advances in the prevention and management of CAD in recent years, the healthcare burden of CAD remains high, driven by population aging, the growing prevalence of metabolic risk factors, and unhealthy lifestyle patterns (4). Early and accurate assessment of CAD severity is therefore essential for risk stratification and individualized clinical management.

The severity of CAD can be assessed using several approaches. Coronary angiography delineates the degree and anatomical distribution of coronary stenosis and underpins several methods of lesion assessment (5). The number of diseased vessels provides a simple estimate of disease extent but does not account for stenosis severity or lesion location (6). The SYNTAX score primarily assesses anatomical complexity, including bifurcation lesions, chronic total occlusions, and calcification, and is used mainly to guide revascularization strategies (7). Fractional flow reserve evaluates the lesion-specific hemodynamic significance of coronary stenosis and identifies ischemia-producing lesions (8). By contrast, the Gensini score quantifies stenosis severity and applies weights based on the perfusion territory and anatomical importance of the affected vessels (9). Higher Gensini scores have also been shown to be closely associated with worse cardiovascular outcomes and an increased risk of major adverse cardiovascular events (10, 11), supporting its clinical relevance for evaluating the severity of CAD. However, calculation of the Gensini score requires invasive coronary angiography. Its use is therefore limited by procedural indications, costs, technical requirements, and potential procedure-related complications. It is therefore unsuitable for large-scale population screening or frequent longitudinal assessment (12). Convenient, efficient, and cost-effective biochemical markers are therefore needed as complementary tools for assessing coronary atherosclerotic burden.

Accumulating evidence links metabolism-related markers to coronary atherosclerotic burden, underscoring the role of metabolic abnormalities in the development and progression of CAD (13). Atherosclerosis results from the long-term interaction of multiple pathophysiological processes, including glucose dysregulation, insulin resistance, lipid metabolism disorders, and other related metabolic abnormalities (14–16). A single metabolic marker may therefore be insufficient to capture an individual’s overall metabolic and atherosclerotic status. The cholesterol-high-density lipoprotein-glucose (CHG) index was recently proposed as a novel composite metabolic marker (17). Emerging evidence associates the CHG index with various adverse metabolic and cardiovascular outcomes (18–21). Studies have also linked the CHG index to carotid atherosclerosis and the risk of in-stent restenosis after percutaneous coronary intervention (PCI) (22, 23). These findings suggest that the index may be useful for comprehensive metabolic risk assessment. However, comparisons with other commonly used metabolic indicators have yielded inconsistent results, and its value requires further evaluation across populations and clinical settings. In patients with CAD, the relationship between the CHG index and coronary atherosclerotic burden remains incompletely understood. Whether the index provides complementary information beyond commonly used metabolic indices is also unclear.

We therefore hypothesized that a higher CHG index would be associated with greater coronary atherosclerotic burden. We evaluated the association between the CHG index and the Gensini score. In exploratory analyses, we also compared its discriminative performance with that of other commonly used metabolic indicators and assessed its incremental value. These analyses may clarify the potential complementary role of the CHG index as a simple, inexpensive, and readily available marker of coronary atherosclerotic burden in patients with CAD.

## 2 Methods

### 2.1 Study design and population

This single-center, retrospective cross-sectional study included consecutive patients with CAD admitted to the First Hospital of Shanxi Medical University between January 2024 and January 2025. CAD was defined as angiographically confirmed diameter stenosis of ≥50% in at least one major epicardial coronary artery—the left main coronary artery, left anterior descending artery, left circumflex artery, or right coronary artery-or one of their major branches, or as a documented history of myocardial infarction (MI) (5).

The inclusion criteria were as follows: (1) age ≥18 years; and (2) angiographically confirmed CAD. The exclusion criteria were: (1) missing data required for calculation of the CHG index, including fasting blood glucose (FBG), total cholesterol (TC), or high-density lipoprotein cholesterol (HDL-C), or missing key clinical covariates; (2) incomplete coronary angiography data precluding accurate assessment of coronary atherosclerotic burden or calculation of the Gensini score; and (3) severe infection, malignancy, severe hepatic or renal dysfunction, or other conditions that could substantially affect glucose–lipid metabolism.

**Figure 1:**
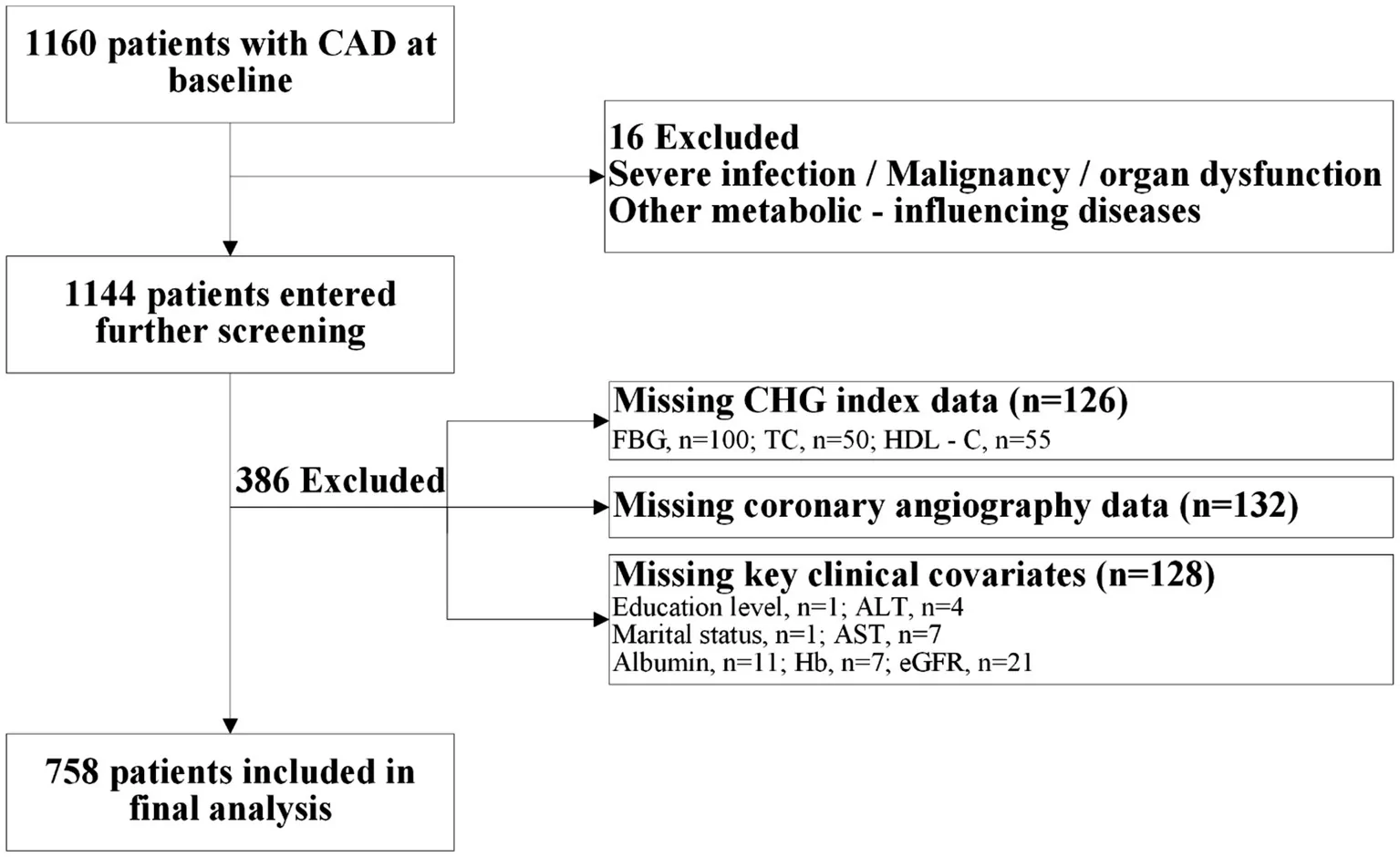
Flowchart of the study sample. CAD, coronary artery disease; CHG index, cholesterol-high-density lipoprotein-glucose index; FBG, fasting blood glucose; TC, total cholesterol; HDL-C, high-density lipoprotein cholesterol; ALT, alanine aminotransferase; AST, aspartate aminotransferase; Hb, hemoglobin; eGFR, estimated glomerular filtration rate.

A total of 1,160 patients were initially screened. Of these, 16 were excluded because of severe infection, malignancy, severe hepatic or renal dysfunction, or other conditions that could substantially affect glucose and lipid metabolism. A further 126 patients were excluded because data required to calculate the CHG index were missing. Another 132 were excluded because coronary angiography data were incomplete or the Gensini score was unavailable. The remaining 128 were excluded because key clinical covariates were missing. Ultimately, 758 patients were included in the final analysis (Figure 1). We compared the baseline characteristics of patients included in the primary analysis with those of patients excluded from the analysis. Overall, the differences in demographic characteristics, comorbidities, acute clinical events, and major metabolic parameters were relatively small (Supplementary Table S1).

The Ethics Committee of the First Hospital of Shanxi Medical University approved the study protocol on March 2, 2023 (No. KYYJ-2023-092). The study was conducted in accordance with the Declaration of Helsinki. Given the retrospective observational design and use of anonymized data, the ethics committee waived the requirement for written informed consent.

### 2.2 Data collection and definitions

Patient data were extracted from the electronic medical record system. Covariates were selected *a priori* based on clinical relevance, previous literature, and data availability.

The variables were grouped as follows:

1. Demographic and lifestyle factors: age, sex, educational level, marital status, smoking status, and drinking status.
2. Anthropometric measurements and vital signs at admission: height, weight, body mass index (BMI), systolic blood pressure (SBP), and diastolic blood pressure (DBP) at admission.
3. Medical history and CAD-related clinical characteristics: hypertension, diabetes, prior PCI, history of MI, lipid-lowering therapy, and glucose-lowering therapy. Hypertension was defined as SBP ≥ 140 mmHg and/or a DBP ≥ 90 mmHg measured on three separate occasions on different days during hospitalization without the use of antihypertensive medications, or a prior diagnosis of hypertension, or current use of antihypertensive medications (24). Diabetes was defined as any of the following: fasting plasma glucose ≥ 7.0 mmol/L during hospitalization, 2-h plasma glucose ≥11.1 mmol/L during an oral glucose tolerance test, glycated hemoglobin ≥6.5%, a prior confirmed diagnosis of diabetes, or current use of glucose-lowering therapy (25). Lipid-lowering and glucose-lowering therapies were defined as documented medication use before admission and were recorded as binary variables because detailed treatment information was unavailable.
4. Laboratory parameters: triglycerides (TG), TC, HDL-C, FBG, low-density lipoprotein cholesterol (LDL-C), alanine aminotransferase (ALT), aspartate aminotransferase (AST), albumin, estimated glomerular filtration rate (eGFR), and hemoglobin (Hb). eGFR was calculated using the Chronic Kidney Disease Epidemiology Collaboration (CKD-EPI) equation. All laboratory measurements used fasting venous blood samples collected on the morning of the second day after admission.
5. Coronary angiographic findings: coronary angiography was performed using standard techniques, and coronary atherosclerotic burden was quantitatively assessed using the Gensini score.

### 2.3 Calculation and categorization of the CHG index and other metabolic markers

The CHG index was calculated using the following formula: CHG index = ln [(TC × FBG) / (2 × HDL-C)], where TC, FBG, and HDL-C were expressed in mg/dL (17). Measurements originally reported in mmol/L were converted to mg/dL using standard conversion factors before calculation. In the primary analyses, the CHG index was examined both as a continuous variable and in quartiles: Q1, ≤4.97; Q2, >4.97 to ≤5.20; Q3, >5.20 to ≤5.51; Q4, >5.51. The lowest quartile (Q1) was used as the reference group. The triglyceride–glucose (TyG) index, atherogenic index of plasma (AIP), and TC/HDL-C ratio were also calculated for comparative analyses. The TyG index was calculated as follows: TyG index = ln [TG (mg/dL) × FBG (mg/dL) / 2] (26). The AIP was calculated using the following formula: AIP = log_10_ (TG / HDL-C) (27), where TG and HDL-C were expressed in the same units. The TC/HDL-C ratio was calculated by dividing TC by HDL-C, with both measurements expressed in the same units.

### 2.4 Calculation and assessment of the Gensini score

Coronary angiography was performed by experienced interventional cardiologists according to standard protocols. All angiographic images were independently reviewed in a blinded manner by two senior interventional cardiologists. In case of disagreement, a third senior interventional cardiologist reviewed the images, and consensus was reached through adjudication. The Gensini score was calculated from coronary angiographic findings and incorporated both the severity of luminal stenosis and the anatomical location of each lesion. Stenoses of 1–25%, 26–50%, 51–75%, 76–90%, 91–99%, and complete occlusion were assigned scores of 1, 2, 4, 8, 16, and 32, respectively. Each severity score was multiplied by a location-specific factor reflecting the functional importance of the affected coronary segment. The weighting factors were 5 for the left main coronary artery and 2.5, 1.5, and 1 for the proximal, mid, and distal left anterior descending artery, respectively. Factors of 1 and 0.5 were used for the first and second diagonal branches, respectively. The factors were 2.5 for the proximal left circumflex artery, 1 for the mid/distal left circumflex artery and posterior descending artery, and 0.5 for the posterolateral branch. A factor of 1 was used for the proximal, mid, and distal right coronary artery and its posterior descending artery. The total Gensini score was the sum of all weighted lesion scores (9). For patients with prior PCI, the score was based on the first angiogram obtained during the index hospitalization. Previously stented segments were assessed according to their current luminal status. Patent stented segments without restenosis received no stenosis score. In-stent restenosis and lesions adjacent to or outside the stented segment were scored using the standard severity and location-specific criteria. Historical pre-PCI lesion severity was not reconstructed. A higher Gensini score indicated a greater current coronary atherosclerotic burden. Because the score was right-skewed, log-transformed (Gensini score + 1) was the primary outcome in the main regression analyses.

### 2.5 Statistical analysis

The Shapiro–Wilk test was used to assess the normality of continuous variables. Participants were categorized into quartiles according to the CHG index (Q1–Q4). Normally distributed continuous variables were expressed as mean ± standard deviation (SD) and compared using one-way analysis of variance (ANOVA). Non-normally distributed continuous variables were presented as median (interquartile range, IQR) and compared using the Kruskal–Wallis test. Categorical variables were expressed as frequencies (percentages) and compared using the chi-square test or Fisher’s exact test, as appropriate.

Multivariable linear regression models were constructed to evaluate the association between the CHG index and log-transformed (Gensini score + 1). The CHG index was analyzed as both a continuous variable and a categorical variable in quartiles, with Q1 serving as the reference group. P for trend was calculated by modeling quartiles as an ordinal variable. Multicollinearity was assessed using variance inflation factors (VIFs) for variables with one degree of freedom. Generalized variance inflation factors (GVIFs) were used for categorical variables with multiple degrees of freedom. For the latter, the adjusted GVIF was calculated as GVIF^(1/[2 × df]). A VIF greater than 10 or an adjusted GVIF greater than √10 indicated potentially problematic multicollinearity. Four progressively adjusted models were constructed: Model 1, unadjusted; Model 2, adjusted for age and sex; Model 3, additionally adjusted for educational level, marital status, BMI, smoking status, drinking status, hypertension, prior PCI, history of MI, lipid-lowering therapy, and glucose-lowering therapy; Model 4, further adjusted for SBP, DBP, ALT, AST, albumin, eGFR, Hb, and LDL-C. Restricted cubic spline (RCS) regression with four knots at the 5th, 35th, 65th, and 95th percentiles was used to examine the potential dose–response relationship between the CHG index and log-transformed (Gensini score + 1). The median CHG value was set as the reference. Overall association and nonlinearity were assessed using the fully adjusted model.

Subgroup analyses were performed stratified by age, sex, BMI, diabetes, hypertension, prior PCI, history of MI, and lipid-lowering therapy. Interaction terms were included to assess effect modification. All subgroup analyses were conducted using the fully adjusted model without further adjustment for the stratification variable. Four sensitivity analyses were conducted: (1) excluding patients with acute MI during the index hospitalization; (2) excluding participants with CHG index values outside the mean ± 3 SD; (3) excluding participants with prior PCI; and (4) repeating the analyses after multiple imputation. Multiple imputation was limited to otherwise eligible patients with an observed Gensini score. Missing CHG components and covariates were imputed by chained equations using 50 datasets and 20 iterations. The CHG index was recalculated in each dataset, and estimates were pooled using Rubin’s rules.

Receiver operating characteristic (ROC) curve analyses were performed to assess the ability of the CHG index and other metabolic markers to discriminate high coronary atherosclerotic burden. High burden was defined as the highest tertile (T3) of the Gensini score (>30) in the present cohort. The apparent stand-alone discriminative performance of each marker was summarized using the area under the curve (AUC) with 95% confidence intervals (CI). A basic clinical model was constructed including age, sex, educational level, marital status, BMI, smoking status, drinking status, hypertension, prior PCI, history of MI, lipid-lowering therapy, glucose-lowering therapy, SBP, DBP, ALT, AST, albumin, eGFR, and Hb. Each metabolic marker was added separately to form an extended model. Model discrimination was assessed using the AUC, and DeLong’s test was used to compare AUCs between models. In addition, continuous net reclassification improvement (NRI), integrated discrimination improvement (IDI), and decision curve analysis (DCA) were used to further evaluate the incremental value of the CHG index. Internal validation was performed using 1,000 bootstrap resamples.

All statistical analyses were performed using R software (version 4.5.2). A two-sided *p* value < 0.05 was considered statistically significant.

## 3 Results

### 3.1 Baseline characteristics stratified by CHG index quartiles

Table 1 presents the characteristics of the 758 participants by CHG index quartile: Q1 (*n* = 190), Q2 (*n* = 189), Q3 (*n* = 189), and Q4 (*n* = 190). Several baseline characteristics differed significantly across quartiles (all *p* < 0.05). Participants in higher quartiles tended to be younger, had a higher BMI, and were more likely to have diabetes and receive glucose-lowering therapy. By contrast, prior PCI, a history of MI, and lipid-lowering therapy were less common in higher quartiles. Higher quartiles were also associated with higher TC, FBG, TG, LDL-C, albumin, eGFR, and Hb levels and higher Gensini scores, whereas HDL-C levels were lower. ALT levels differed significantly across quartiles, although no clear monotonic trend was evident. Sex, educational level, marital status, smoking status, drinking status, hypertension, and AST levels did not differ significantly across quartiles (all *p* > 0.05).

**Table 1:** Baseline characteristics of the study population according to quartiles of the CHG index.

Variables	Total population	Q1	Q2	Q3	Q4	*p* value
*N*	758	190	189	189	190	
Age, years	58.98 ± 9.04	61.43 ± 7.74	59.02 ± 9.64	58.21 ± 9.15	57.27 ± 9.06	<0.001
Sex, *n* (%)						0.931
Male	573 (75.6%)	143 (75.3%)	144 (76.2%)	140 (74.1%)	146 (76.8%)	
Female	185 (24.4%)	47 (24.7%)	45 (23.8%)	49 (25.9%)	44 (23.2%)	
BMI, kg/m^2^	25.83 ± 3.43	24.55 ± 3.63	25.98 ± 2.97	26.12 ± 3.41	26.69 ± 3.33	<0.001
Educational level, *n* (%)						0.774
Junior or below	372 (49.1%)	103 (54.2%)	89 (47.1%)	91 (48.1%)	89 (46.8%)	
High School or Technical	161 (21.2%)	39 (20.5%)	41 (21.7%)	41 (21.7%)	40 (21.1%)	
College or above	225 (29.7%)	48 (25.3%)	59 (31.2%)	57 (30.2%)	61 (32.1%)	
Marital status, *n* (%)						0.522
Married	735 (97.0%)	182 (95.8%)	185 (97.9%)	185 (97.9%)	183 (96.3%)	
Other	23 (3.0%)	8 (4.2%)	4 (2.1%)	4 (2.1%)	7 (3.7%)	
Smoking status, *n* (%)						0.451
Never	346 (45.6%)	86 (45.3%)	93 (49.2%)	81 (42.9%)	86 (45.3%)	
Current	287 (37.9%)	73 (38.4%)	59 (31.2%)	78 (41.3%)	77 (40.5%)	
Former	125 (16.5%)	31 (16.3%)	37 (19.6%)	30 (15.9%)	27 (14.2%)	
Drinking status, *n* (%)						0.329
Never	462 (60.9%)	128 (67.4%)	111 (58.7%)	110 (58.2%)	113 (59.5%)	
Current	234 (30.9%)	48 (25.3%)	62 (32.8%)	59 (31.2%)	65 (34.2%)	
Former	62 (8.2%)	14 (7.4%)	16 (8.5%)	20 (10.6%)	12 (6.3%)	
Hypertension, *n* (%)						0.066
Yes	433 (57.1%)	97 (51.1%)	120 (63.5%)	113 (59.8%)	103 (54.2%)	
No	325 (42.9%)	93 (48.9%)	69 (36.5%)	76 (40.2%)	87 (45.8%)	
Diabetes, *n* (%)						<0.001
Yes	198 (26.1%)	25 (13.2%)	31 (16.4%)	48 (25.4%)	94 (49.5%)	
No	560 (73.9%)	165 (86.8%)	158 (83.6%)	141 (74.6%)	96 (50.5%)	
Prior PCI, *n* (%)						<0.001
Yes	191 (25.2%)	73 (38.4%)	49 (25.9%)	34 (18.0%)	35 (18.4%)	
No	567 (74.8%)	117 (61.6%)	140 (74.1%)	155 (82.0%)	155 (81.6%)	
History of MI, *n* (%)						0.014
Yes	97 (12.8%)	34 (17.9%)	29 (15.3%)	16 (8.5%)	18 (9.5%)	
No	661 (87.2%)	156 (82.1%)	160 (84.7%)	173 (91.5%)	172 (90.5%)	
Lipid-lowering therapy, *n* (%)						<0.001
Yes	309 (40.8%)	104 (54.7%)	93 (49.2%)	60 (31.7%)	52 (27.4%)	
No	449 (59.2%)	86 (45.3%)	96 (50.8%)	129 (68.3%)	138 (72.6%)	
Glucose-lowering therapy, *n* (%)						<0.001
Yes	199 (26.3%)	25 (13.2%)	31 (16.4%)	49 (25.9%)	94 (49.5%)	
No	559 (73.7%)	165 (86.8%)	158 (83.6%)	140 (74.1%)	96 (50.5%)	
TC, mmol/L	3.73 (3.19, 4.62)	3.19 (2.71, 3.69)	3.49 (3.10, 4.21)	3.97 (3.47, 4.69)	4.59 (3.82, 5.32)	<0.001
HDL-C, mmol/L	0.98 (0.86, 1.14)	1.08 (0.95, 1.32)	1.01 (0.88, 1.14)	0.92 (0.82, 1.07)	0.92 (0.83, 1.07)	<0.001
FBG, mmol/L	5.17 (4.65, 6.22)	4.61 (4.28, 5.00)	4.99 (4.63, 5.43)	5.30 (4.84, 6.02)	6.95 (5.80, 8.96)	<0.001
TG, mmol/L	1.46 (1.09, 2.07)	1.05 (0.84, 1.35)	1.34 (1.03, 1.73)	1.63 (1.28, 2.24)	2.12 (1.54, 3.00)	<0.001
LDL-C, mmol/L	2.32 (1.93, 2.92)	1.90 (1.57, 2.18)	2.20 (1.92, 2.65)	2.59 (2.10, 3.07)	2.92 (2.46, 3.55)	<0.001
ALT, U/L	22.00 (16.00, 32.00)	21.00 (15.00, 31.00)	20.00 (16.00, 30.00)	22.00 (15.00, 32.00)	25.00 (18.00, 36.00)	0.002
AST, U/L	21.00 (17.00, 26.00)	21.50 (18.00, 26.00)	20.00 (17.00, 24.00)	20.00 (17.00, 27.00)	22.00 (17.00, 29.75)	0.141
Albumin, g/L	41.15 ± 3.34	40.12 ± 3.23	41.18 ± 3.36	41.44 ± 3.38	41.87 ± 3.15	<0.001
eGFR, mL/min/1.73 m^2^	98.63 ± 11.97	97.52 ± 12.04	97.83 ± 11.10	98.23 ± 11.10	100.94 ± 13.29	0.020
Hb, g/L	144.16 ± 14.43	140.31 ± 12.99	143.89 ± 13.92	144.78 ± 15.08	147.67 ± 14.76	<0.001
Gensini score	20.00 (10.00, 37.75)	16.00 (9.00, 31.50)	19.00 (9.00, 34.00)	20.00 (10.00, 36.00)	27.00 (12.00, 50.00)	<0.001

CHG index, cholesterol-high-density lipoprotein-glucose index; BMI, body mass index; PCI, percutaneous coronary intervention; MI, myocardial infarction; TC, total cholesterol; HDL-C, high-density lipoprotein cholesterol; FBG, fasting blood glucose; TG, triglycerides; LDL-C, low-density lipoprotein cholesterol; ALT, alanine aminotransferase; AST, aspartate aminotransferase; eGFR, estimated glomerular filtration rate; Hb, hemoglobin.

### 3.2 Association between the CHG index and log-transformed (Gensini score + 1): multivariable linear regression analysis

**Figure 2:**
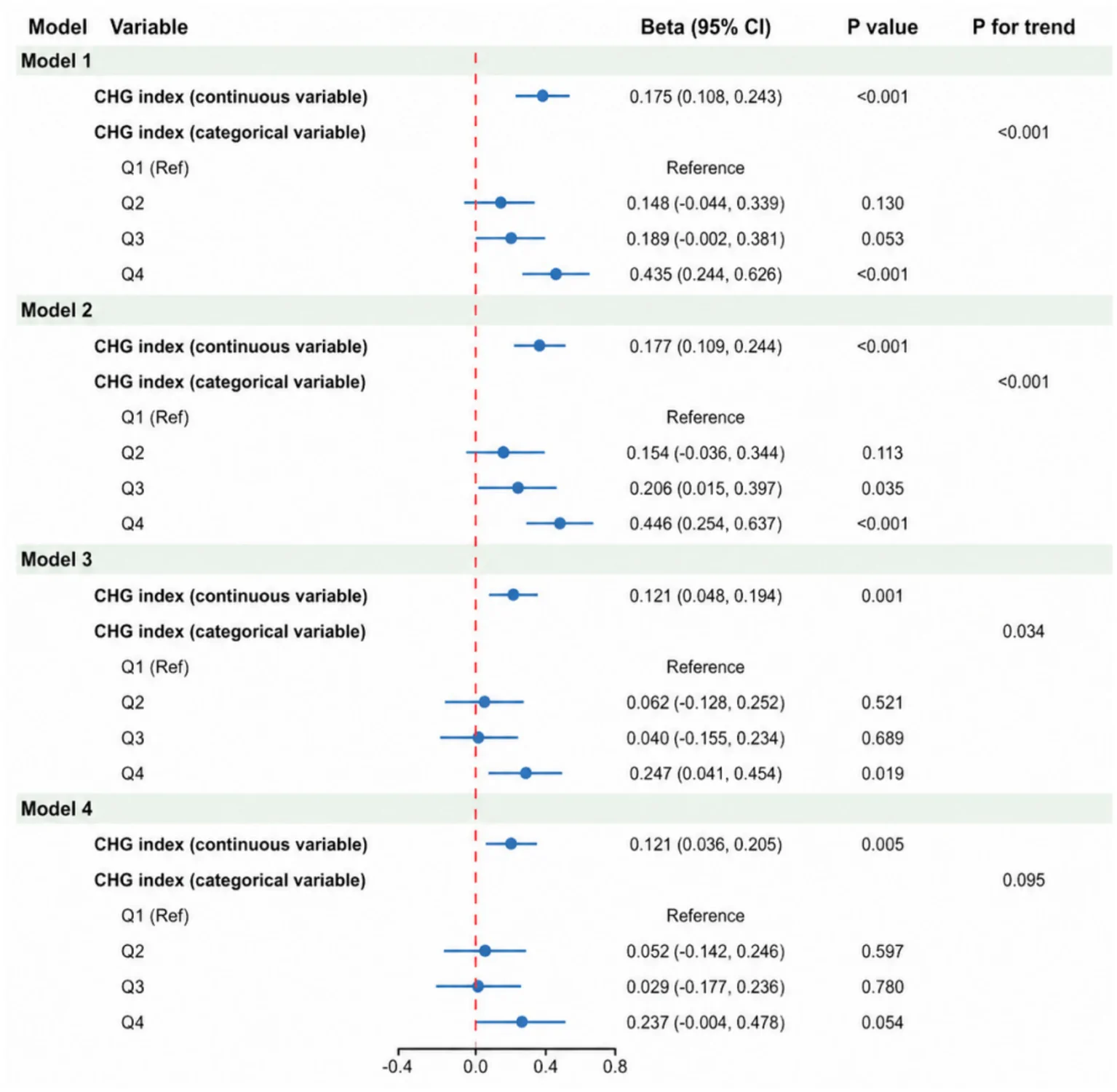
Association between the CHG index and log-transformed (Gensini score + 1) in multivariable linear regression models. Model 1: Unadjusted; Model 2: Adjusted for sex and age; Model 3: Further adjusted for educational level, marital status, BMI, smoking status, drinking status, hypertension, prior PCI, history of MI, lipid-lowering therapy, and glucose-lowering therapy. Model 4: Further adjusted for SBP, DBP, ALT, AST, albumin, eGFR, Hb, and LDL-C. *β* coefficients represent the change in log-transformed (Gensini score + 1) per 1-*SD* increase in the CHG index. Q1 was used as the reference group. CHG index, cholesterol-high-density lipoprotein-glucose index; CI: Confidence interval; BMI, body mass index; PCI, percutaneous coronary intervention; MI, myocardial infarction; SBP, systolic blood pressure; DBP, diastolic blood pressure; ALT, alanine aminotransferase; AST, aspartate aminotransferase; eGFR, estimated glomerular filtration rate; Hb, Hemoglobin; LDL-C, low-density lipoprotein cholesterol; SD, standard deviation.

As shown in Figure 2, the standardized CHG index was positively associated with log-transformed (Gensini score + 1) across all progressively adjusted models. In the fully adjusted model (Model 4), each 1-SD increase in the CHG index was associated with a 0.121-unit increase in log-transformed (Gensini score + 1) (*β* = 0.121, 95% CI: 0.036–0.205, *p* = 0.005). In the quartile-based analysis, participants in the highest CHG index quartile had significantly higher log-transformed (Gensini score + 1) values than those in the lowest quartile in Model 3 (*β* = 0.247, 95% CI: 0.041–0.454, *p* = 0.019). After further adjustment in Model 4, however, the association was attenuated and no longer reached statistical significance (β = 0.237, 95% CI: −0.004 to 0.478, *p* = 0.054). The linear trend across CHG index quartiles was also not statistically significant in the fully adjusted model (P for trend = 0.095). Overall, the CHG index remained significantly associated with coronary atherosclerotic burden when modeled as a standardized continuous variable, whereas the quartile-based association was attenuated after full adjustment. There was no evidence of substantial multicollinearity in Model 4. The highest conventional VIF was 2.242 for prior PCI, whereas the VIF for the CHG index was 1.745. Among categorical variables with more than 1 degree of freedom, smoking status had the highest adjusted GVIF of 1.242. Detailed multicollinearity diagnostics are provided in Supplementary Table S2.

### 3.3 Dose–response relationship between the CHG index and log-transformed (Gensini score + 1)

**Figure 3:**
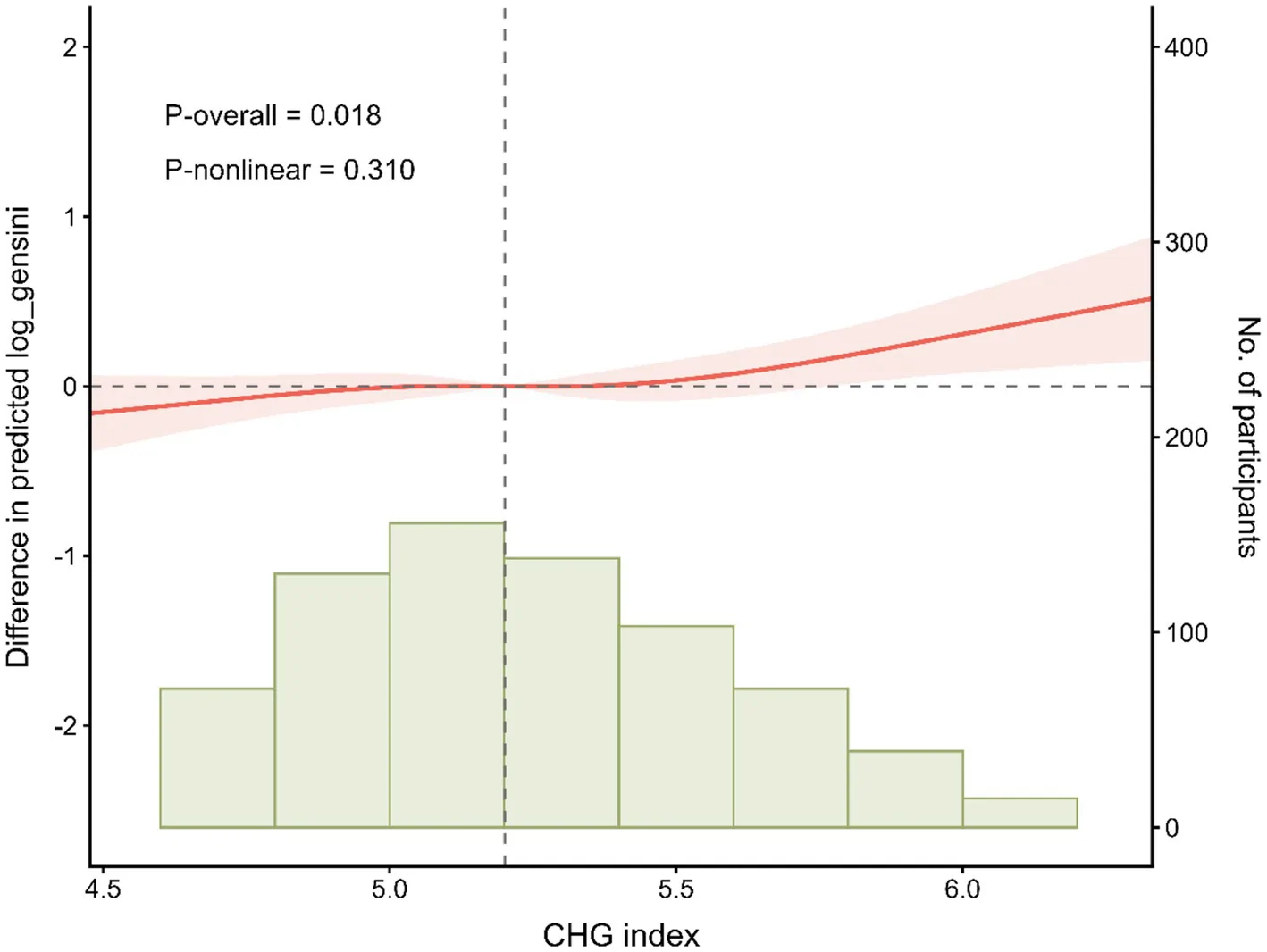
RCS of the association between the CHG index and log-transformed (Gensini score + 1). The solid line represents the estimated association, and the shaded area indicates the 95% CI. The histogram shows the distribution of the CHG index. The median CHG value was used as the reference. RCS, restricted cubic spline; CHG index, cholesterol-high-density lipoprotein-glucose index; CI: Confidence interval.

RCS analysis based on the fully adjusted model was used to assess the association between the CHG index and log-transformed (Gensini score + 1). As shown in Figure 3, the overall association was significant (P for overall association = 0.018), with no evidence of nonlinearity (P for nonlinearity = 0.310). The spline curve showed a generally increasing pattern of log-transformed (Gensini score + 1) at higher CHG levels. These findings indicated an approximately linear association between the CHG index and coronary atherosclerotic burden.

### 3.4 Subgroup analysis

**Figure 4:**
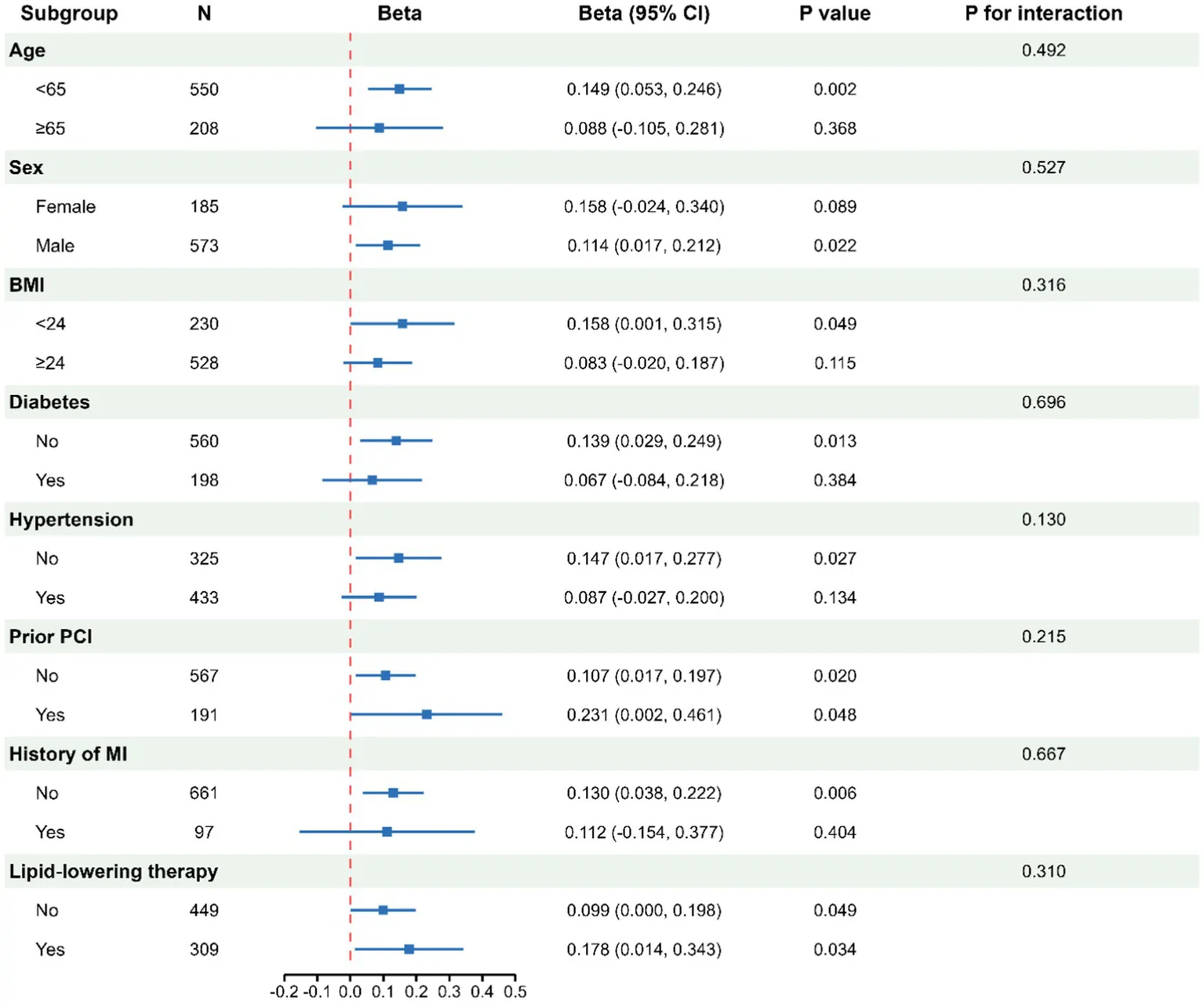
Subgroup analyses. Forest plot showing the association of each 1-SD increase in the CHG index with log-transformed (Gensini score + 1) across predefined subgroups. *p* values for interaction were obtained from multiplicative interaction terms in the linear regression models. CHG index, cholesterol-high-density lipoprotein-glucose index; SD, standard deviation; CI: Confidence interval; PCI, percutaneous coronary intervention; MI, myocardial infarction.

Prespecified subgroup analyses were conducted by age, sex, BMI, diabetes, hypertension, prior PCI, history of MI, and lipid-lowering therapy. As shown in Figure 4, effect estimates were generally positive across subgroups, although some associations did not reach statistical significance. No significant interactions were observed for any stratification variable (all P for interaction > 0.05). Thus, there was no clear evidence of effect modification of the linear association. Similarly, subgroup-specific RCS analyses showed no significant differences in overall exposure–response curves between strata (all P for interaction > 0.05; Supplementary Figure S1).

### 3.5 Sensitivity analyses

The positive association between the CHG index and log-transformed (Gensini score + 1) remained robust in all four sensitivity analyses. The multiple-imputation analysis included 1,000 eligible patients with an observed Gensini score. A higher CHG index remained significantly associated with higher log-transformed (Gensini score + 1) (*β* = 0.132, 95% CI, 0.058–0.206; *p* < 0.001). Similar results were observed after excluding patients with a history of PCI (*β* = 0.107, 95% CI, 0.017–0.198; *p* = 0.020). Results were also consistent after excluding CHG index values beyond the mean ± 3 SD (*β* = 0.121, 95% CI, 0.024–0.217; *p* = 0.014). Finally, the results remained consistent after excluding patients with acute MI during the index hospitalization (*β* = 0.094, 95% CI, 0.003–0.185; *p* = 0.045). These findings were directionally consistent with the primary analysis, supporting the robustness of the association (Supplementary Table S3).

### 3.6 Comparison of the discriminative ability of the CHG index and other metabolic markers for high coronary atherosclerotic burden

**Figure 5:**
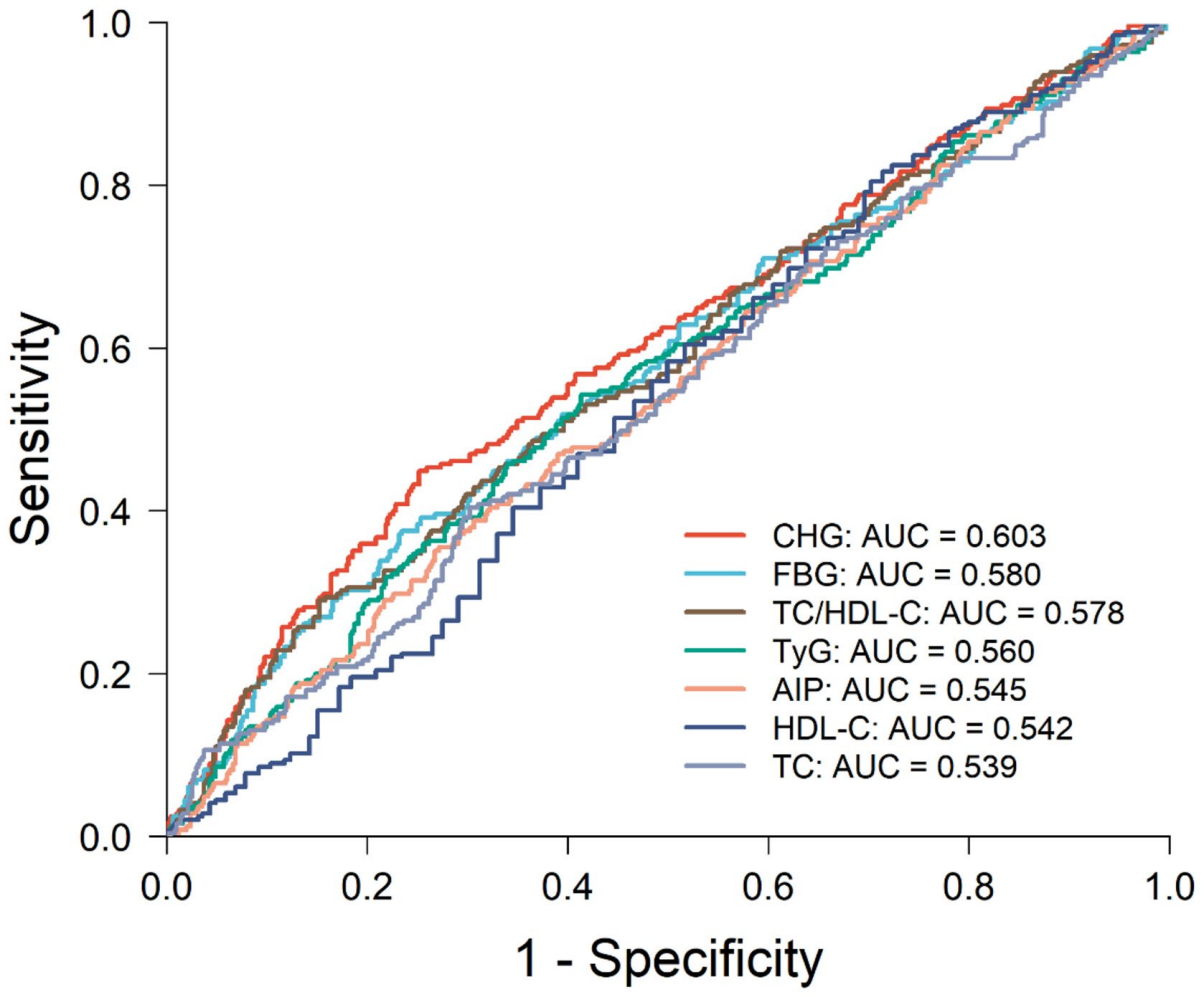
ROC curves comparing the CHG index and other metabolic markers for discriminating high coronary atherosclerotic burden. ROC, receiver operating characteristic; CHG index, cholesterol-high-density lipoprotein-glucose index; AUC, area under the curve; FBG, fasting blood glucose; TyG index, triglyceride-glucose index; HDL-C, high-density lipoprotein cholesterol; AIP, atherogenic index of plasma; TC, total cholesterol.

As shown in Figure 5, the CHG index had the highest AUC for discriminating high coronary atherosclerotic burden among the evaluated metabolic markers. However, its overall discriminative ability was modest (AUC = 0.603). The corresponding AUCs for FBG, the TC/HDL-C ratio, the TyG index, AIP, HDL-C, and TC were 0.580, 0.578, 0.560, 0.545, 0.542, and 0.539, respectively. In paired DeLong comparisons, the AUC of the CHG index was significantly higher than those of the TyG index (*p* = 0.005), AIP (*p* = 0.004), HDL-C (*p* = 0.023), and TC (*p* = 0.003). The CHG index did not differ significantly from FBG (*p* = 0.198) or the TC/HDL-C ratio (*p* = 0.096).

### 3.7 Incremental discriminative value of the CHG index for high coronary atherosclerotic burden

**Figure 6:**
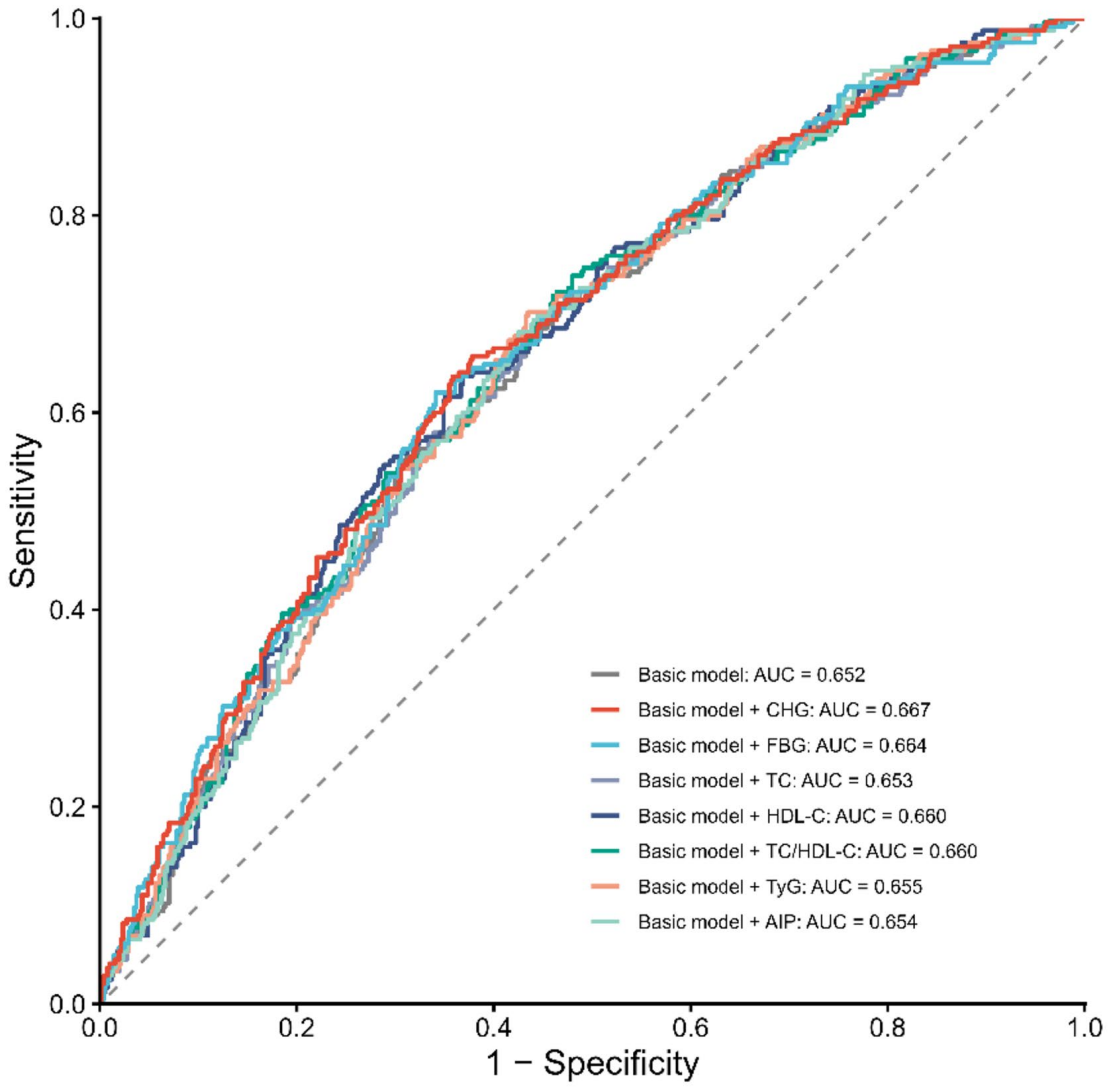
Apparent ROC curves for identifying high coronary atherosclerotic burden. ROC, Receiver operating characteristic; CHG index, Cholesterol-high-density lipoprotein-glucose index; FBG, Fasting blood glucose; TC, Total cholesterol; HDL-C, High-density lipoprotein cholesterol; TyG index, Triglyceride-glucose index; AIP, Atherogenic index of plasma; AUC, Area under the curve.

Among the four progressively adjusted models, Model 4 had the highest apparent AUC for discriminating high coronary atherosclerotic burden (AUC = 0.666, 95% CI, 0.626–0.707; Supplementary Figure S2). When each metabolic marker was added separately to the basic model, the extended model that included the CHG index had the numerically highest apparent AUC (Figure 6). Specifically, the apparent AUC increased from 0.652 for the basic model to 0.667 after adding the CHG index. However, the difference was not statistically significant according to DeLong’s test (*p* = 0.168). Adding the CHG index yielded a significant continuous NRI of 0.135 (95% CI, 0.006–0.364, *p* = 0.042). It also yielded an IDI of 0.016 (95% CI, 0.003–0.037, *p* = 0.002). DCA showed that adding the CHG index modestly improved net benefit over a limited range of intermediate threshold probabilities (Supplementary Figure S3A). After internal validation using 1,000 bootstrap resamples, optimism-corrected AUCs were generally lower than the corresponding apparent estimates. Nevertheless, the model that included the CHG index retained the numerically highest optimism-corrected AUC among the extended models. Bootstrap-corrected discrimination and calibration metrics are presented in Supplementary Table S4. Incremental discrimination and reclassification results are provided in Supplementary Table S5. The optimism-corrected decision curves are shown in Supplementary Figure S3B.

## 4 Discussion

In patients with CAD, we found that a higher CHG index was associated with greater coronary atherosclerotic burden and an approximately linear dose–response relationship. The association was directionally consistent across subgroups, with no significant interactions, although some subgroup-specific associations were not statistically significant. Sensitivity analyses yielded consistent results. Among the metabolic markers evaluated, the CHG index demonstrated the numerically highest stand-alone discriminative performance for high coronary atherosclerotic burden, although its overall discriminative ability remained modest. When the CHG index was added to the basic model, the model AUC increased slightly, and the improvement did not reach statistical significance. Nevertheless, the CHG index-extended model had the numerically highest AUC among all extended models, and both continuous NRI and IDI were statistically significant. DCA further indicated a modest net clinical benefit over a limited range of intermediate threshold probabilities. After internal validation, the CHG index-extended model retained the numerically highest optimism-corrected AUC, and the overall direction of the findings remained consistent.

Across CHG index groups, patients with higher CHG levels were younger and had lower prevalences of prior PCI and MI. This pattern may be related to the restriction of the study population to patients with established CAD. In this population, younger patients may represent a subgroup in whom non-age-related factors contributed more strongly to the earlier development of CAD. Such factors include abnormalities in glucose and lipid metabolism. Diabetes, insulin resistance, obesity, hypertriglyceridemia, and low HDL-C levels are particularly associated with premature CAD or acute coronary syndrome (28, 29). These associations may partly explain the higher CHG index levels among younger patients in the present study. Conversely, patients with prior cardiovascular events or PCI are generally more likely to have received intensive secondary prevention measures, including lipid-lowering therapy, lifestyle interventions, and management of other cardiometabolic risk factors (5). These interventions may have altered the components of the CHG index measured at study enrollment. Therefore, the inverse associations of prior MI and PCI with the CHG index may partly reflect treatment-related confounding and reverse causation (30). These findings should not be interpreted as indicating that older age or previous cardiovascular events confer metabolic protection.

Recent studies have associated the CHG index with CVD, metabolic disorders, and adverse clinical outcomes (31–36). Higher CHG index levels have also been associated with more severe vascular lesions (22, 23, 37). Building on these findings, our results showed a positive association between the CHG index and Gensini score among patients with CAD. The dose–response relationship was approximately linear. However, previous studies have reported heterogeneous dose–response patterns between the CHG index and clinical outcomes. For example, cohort studies by Wei et al. (38), Zhu et al. (19), and Tian et al. (21) reported nonlinear U-shaped or J-shaped associations with all-cause mortality or long-term survival. The “lipid paradox” or competing risks at extremely low glucose–lipid levels may partly explain these patterns. Very low biomarker levels may indicate malnutrition, frailty, or chronic wasting rather than cardiovascular protection, thereby increasing all-cause mortality (39). By contrast, studies of specific cardiovascular events or structural vascular disease are less affected by terminal disease states and competing risks. In these settings, the CHG index has more consistently shown a positive linear association (22, 32, 40). Our findings for coronary atherosclerotic burden are generally consistent with this evidence. In the quartile analysis, effect estimates generally increased across CHG index quartiles, but the association between the highest and lowest quartiles was attenuated after full adjustment. On the one hand, categorizing continuous variables may lead to information loss and reduced statistical power (41). On the other hand, all participants had established CAD and therefore a generally high baseline risk, which may have attenuated differences between intermediate groups. Liu et al. reported a similar attenuation of the risk gradient among individuals with pre-existing cardiometabolic disease (18). Continuous-variable and RCS analyses may therefore have used the information in the CHG index more fully. Nevertheless, the weaker categorical findings indicate that the magnitude of the association should not be overstated.

Notably, the regression coefficient for the CHG index decreased from 0.177 in Model 2 to 0.121 in Model 3. This attenuation suggests that underlying metabolic abnormalities may partly account for the association between the CHG index and coronary atherosclerotic burden. Disturbances in glucose metabolism may be particularly important. Substantial epidemiological and clinical evidence has established diabetes as a major independent risk factor for CAD. Broader disturbances in glucose metabolism are also associated with increased cardiovascular risk and contribute to the initiation and progression of atherosclerosis (42, 43). Mechanistically, hyperglycemia and insulin resistance can promote endothelial dysfunction, oxidative stress, and inflammatory responses. They can also promote glycation of atherogenic lipoproteins, thereby accelerating foam-cell formation and coronary plaque progression (44, 45). However, atherosclerosis is a complex, multifactorial process in which lipid abnormalities also have an important role. These metabolic disturbances may interact and reinforce one another, jointly contributing to the development and progression of atherosclerosis (46). Several metabolic indices have been associated with CAD severity. These include the TyG index, a commonly used surrogate marker of insulin resistance; AIP, an indicator of an atherogenic lipid profile; and the TC/HDL-C ratio, which reflects the balance between atherogenic cholesterol burden and HDL-related protection (47–49). An increased cholesterol burden promotes the retention of atherogenic lipoproteins within the arterial wall (50, 51). Reduced HDL-C levels or impaired HDL function may weaken reverse cholesterol transport and the antioxidant and anti-inflammatory vascular effects of HDL (52, 53). Together, these glucose- and lipid-related abnormalities may promote lipid deposition and vascular injury. Unlike indices that capture a single metabolic dimension, the CHG index integrates TC, HDL-C, and FBG. It therefore combines information about glucose metabolism, cholesterol burden, and HDL-mediated vascular protection and may better capture composite metabolic disturbance.

We found that the fully adjusted model, which additionally incorporated laboratory variables including eGFR, achieved the highest AUC. This finding suggests that assessing coronary atherosclerotic burden may require a broader multisystem perspective beyond conventional cardiovascular risk factors. Previous studies have associated impaired kidney function with higher Gensini scores and greater coronary atherosclerotic burden (54, 55). These findings underscore the close interrelationship among CAD, kidney dysfunction, and metabolic abnormalities. In 2023, the American Heart Association formally introduced and systematically characterized cardiovascular–kidney–metabolic (CKM) syndrome. Rather than the simple coexistence of CAD, kidney disease, and metabolic dysfunction, this framework emphasizes their pathophysiological interconnections (56). Hyperglycemia, insulin resistance, and dyslipidemia may promote atherosclerosis through inflammation, oxidative stress, endothelial dysfunction, and vascular injury while also accelerating kidney dysfunction. Impaired kidney function may, in turn, worsen blood pressure dysregulation, inflammation, and vascular dysfunction, amplifying cardiovascular injury in a self-reinforcing cycle (57, 58). In our study, the CHG index captured abnormalities in glucose and lipid metabolism, eGFR reflected kidney function, and conventional risk factors characterized the underlying cardiovascular context. Integrating these domains may characterize the CKM-related multisystem risk profile in patients with CAD more comprehensively than any single marker. This approach may also identify cumulative risk that would otherwise be overlooked. Moreover, the association between the CHG index and coronary atherosclerotic burden persisted after additional adjustment that included eGFR. The metabolic information captured by the CHG index was therefore not fully explained by kidney function, and the two measures may provide complementary rather than interchangeable information.

This study has several strengths. First, it was conducted in a real-world clinical setting and included patients with angiographically confirmed CAD, providing high diagnostic certainty. Second, coronary atherosclerotic burden was assessed using the Gensini score. This score incorporates the degree of luminal stenosis and the anatomical importance of affected coronary segments, providing greater detail than binary disease definitions or vessel-based classifications. Third, we analyzed the CHG index as both a continuous and categorical variable and used RCS models to explore the dose–response relationship. We also conducted subgroup and sensitivity analyses to assess the robustness of the findings. Finally, exploratory analyses directly compared the discriminative performance and incremental value of the CHG index with those of other metabolic markers. Internal validation used bootstrap resampling. Together, these approaches enabled a comprehensive and robust evaluation of the relationship between the CHG index and coronary atherosclerotic burden in patients with CAD.

Several limitations should also be acknowledged. First, the single-center, retrospective cross-sectional design precluded causal inference. Second, the study included only hospitalized patients with angiographically confirmed CAD and excluded those with missing key data. This selection may have introduced bias and limited the generalizability of the findings. Third, the CHG index was based on a single measurement at admission and therefore did not reflect long-term exposure or temporal variability. Fourth, information was unavailable on medication dosage, treatment duration and adherence, lifestyle factors, inflammatory markers, disease duration, and long-term glycemic and blood pressure control. This limitation may have resulted in residual confounding. Finally, the Gensini score quantifies only the anatomical severity of coronary stenosis. It does not characterize plaque composition, plaque stability, or functional ischemia and may underestimate cumulative coronary disease burden in patients with prior PCI.

## 5 Conclusion

Among patients with angiographically confirmed CAD, a higher CHG index was associated with greater coronary atherosclerotic burden and an approximately linear dose–response relationship. Exploratory analyses further suggested that the CHG index may provide some incremental value beyond conventional clinical factors. As a simple, low-cost, and readily available composite marker of glucose–lipid metabolism, the CHG index may provide complementary metabolic information for assessing coronary atherosclerotic burden. Large, multicenter prospective studies are needed to validate its longitudinal dynamics and prognostic value for long-term cardiovascular outcomes.

## Data Availability

The raw data supporting the conclusions of this article will be made available by the authors, without undue reservation.

## Glossary

CHG: Cholesterol-high-density lipoprotein-glucose
CVD: Cardiovascular disease
CAD: Coronary artery disease
PCI: Percutaneous coronary intervention
FBG: Fasting blood glucose
TC: Total cholesterol
HDL-C: High-density lipoprotein cholesterol
LDL-C: low-density lipoprotein cholesterol
MI: Myocardial infarction
BMI: Body mass index
SBP: Systolic blood pressure
DBP: Diastolic blood pressure
TG: Triglycerides
ALT: Alanine aminotransferase
AST: Aspartate aminotransferase
eGFR: Estimated glomerular filtration rate
Hb: hemoglobin
TyG: Triglyceride–glucose index
AIP: Atherogenic index of plasma
RCS: Restricted cubic spline
ROC: Receiver operating characteristic
AUC: Area under the curve
NRI: Net reclassification improvement
IDI: Integrated discrimination improvement
DCA: Decision curve analysis
CI: confidence interval
SD: standard deviation
