# Supplementary material for: Association of the cholesterol-high-density lipoprotein-glucose index with coronary atherosclerotic burden in patients with coronary artery disease
Source: Front Nutr. 2026 Aug 25;13:1906236. doi: 10.3389/fnut.2026.1906236 (PMC13549997; doi:10.3389/fnut.2026.1906236)
Supplement: Supplementary file 1 [file Supplementary_file_1.DOCX]

**Supplementary Table S1.** Comparison of baseline characteristics between patients included in and excluded from the primary analysis

| Variables | Overall | Included | Excluded | P value |
| --- | --- | --- | --- | --- |
| N | 1160 | 758 | 402 |  |
| Age, years | 58.87 ± 9.30 | 58.98 ± 9.04 | 58.66 ± 9.78 | 0.582 |
| Sex, n (%) |  |  |  | 0.842 |
| Male | 879 (75.8) | 573 (75.6%) | 306 (76%) |  |
| Female | 281 (24.2) | 185 (24.4%) | 96 (24%) |  |
| BMI, kg/m^2^ | 25.96 ± 3.38 | 25.83 ± 3.43 | 26.20 ± 3.27 | 0.075 |
| Educational level, n (%) |  |  |  | 0.040 |
| Junior or below | 552 (47.6) | 372 (49.1%) | 180 (45%) |  |
| High School or Technical | 234 (20.2) | 161 (21.2%) | 73 (18%) |  |
| College or above | 373 (32.2) | 225 (29.7%) | 148 (37%) |  |
| Marital status, n (%) |  |  |  | 0.674 |
| Married | 1122 (96.7) | 735 (97.0%) | 387 (97%) |  |
| Other | 37 (3.2) | 23 (3.0%) | 14 (3.5%) |  |
| Smoking status, n (%) |  |  |  | 0.401 |
| Never | 524 (45.2) | 346 (45.6%) | 178 (44%) |  |
| Current | 432 (37.2) | 287 (37.9%) | 145 (36%) |  |
| Former | 204 (17.6) | 125 (16.5%) | 79 (20%) |  |
| Drinking status, n (%) |  |  |  | 0.246 |
| Never | 715 (61.6) | 462 (60.9%) | 253 (63%) |  |
| Current | 342 (29.5) | 234 (30.9%) | 108 (27%) |  |
| Former | 103 (8.9) | 62 (8.2%) | 41 (10%) |  |
| Hypertension, n (%) |  |  |  | 0.495 |
| Yes | 671 (57.8) | 433 (57.1%) | 238 (59%) |  |
| No | 489 (42.2) | 325 (42.9%) | 164 (41%) |  |
| Diabetes, n (%) |  |  |  | 0.206 |
| Yes | 317 (27.3) | 198 (26.1%) | 119 (30%) |  |
| No | 843 (72.7) | 560 (73.9%) | 283 (70%) |  |
| Acute MI, n (%) | 121 (10.4) | 82 (10.8) | 39 (9.7) | 0.554 |
| TC, mmol/L | 3.69 (3.12, 4.52) | 3.73 (3.19, 4.62) | 3.59 (3.01, 4.33) | 0.300 |
| HDL-C, mmol/L | 0.98 (0.86, 1.14) | 0.98 (0.86, 1.14) | 0.99 (0.85, 1.14) | 0.966 |
| FBG, mmol/L | 5.18 (4.68, 6.13) | 5.17 (4.65, 6.22) | 5.21 (4.73, 6.02) | 0.901 |
| TG, mmol/L | 1.44 (1.06, 2.02) | 1.46 (1.09, 2.07) | 1.39 (1.04, 1.93) | 0.105 |
| ALT, U/L | 22.00 (16.00, 33.00) | 22.00 (16.00, 32.00) | 23.00 (16.00, 35.00) | 0.063 |
| AST, U/L | 21.00 (17.00, 27.00) | 21.00 (17.00, 26.00) | 22.00 (18.00, 28.00) | 0.179 |
| Albumin, g/L | 41.20 ± 3.44 | 41.15 ± 3.34 | 41.35 ± 3.78 | 0.490 |
| eGFR, mL/min/1.73m^2^ | 98.23 ± 12.34 | 98.63 ± 11.97 | 97.36 ± 13.05 | 0.121 |
| Hb, g/L | 143.95 ± 14.58 | 144.16 ± 14.43 | 143.53 ± 14.89 | 0.494 |

BMI, body mass index; MI, myocardial infarction; TC, total cholesterol; HDL-C, high-density lipoprotein cholesterol; FBG, fasting blood glucose; TG, triglycerides; ALT, alanine aminotransferase; AST, aspartate aminotransferase; eGFR, estimated glomerular filtration rate; Hb, hemoglobin.

**Supplementary Table S2.** Multicollinearity diagnostics for the fully adjusted model (Model 4)

| Variable | Df | VIF/GVIF | Adjusted GVIF |
| --- | --- | --- | --- |
| CHG index | 1 | 1.745 | – |
| Age | 1 | 1.939 | – |
| Sex | 1 | 1.918 | – |
| BMI | 1 | 1.233 | – |
| Marital status | 1 | 1.037 | – |
| Educational level | 2 | 1.115 | 1.028 |
| Smoking status | 2 | 2.381 | 1.242 |
| Drinking status | 2 | 1.925 | 1.178 |
| Hypertension | 1 | 1.192 | – |
| Prior PCI | 1 | 2.242 | – |
| History of MI | 1 | 1.322 | – |
| Lipid-lowering therapy | 1 | 2.152 | – |
| Glucose-lowering therapy | 1 | 1.275 | – |
| SBP | 1 | 1.663 | – |
| DBP | 1 | 1.750 | – |
| ALT | 1 | 1.489 | – |
| AST | 1 | 1.425 | – |
| Albumin | 1 | 1.276 | – |
| eGFR | 1 | 1.549 | – |
| Hb | 1 | 1.674 | – |
| LDL-C | 1 | 1.751 | – |

VIF, variance inflation factor; GVIF, generalized variance inflation factor; Df, degrees of freedom; CHG index, cholesterol-high-density lipoprotein-glucose index; BMI, body mass index; PCI, percutaneous coronary intervention; MI, myocardial infarction; SBP, systolic blood pressure; DBP, diastolic blood pressure; ALT, alanine aminotransferase; AST, aspartate aminotransferase; eGFR, estimated glomerular filtration rate; Hb, hemoglobin; LDL-C, low-density lipoprotein cholesterol.

**Supplementary Figure S1**. Subgroup-specific RCS analyses of the association between the CHG index and log-transformed (Gensini score + 1)

Panels A–H show subgroup-specific RCS analyses stratified by sex, age, hypertension, prior PCI, diabetes, history of MI, BMI, and lipid-lowering therapy. Solid lines represent estimated associations, shaded areas indicate 95% CIs, and histograms show the CHG index distribution. The vertical dashed line denotes the median CHG index, and P for interaction were derived from fully adjusted RCS models.
CHG index, cholesterol-high-density lipoprotein-glucose index; RCS, restricted cubic spline; CI, confidence interval; BMI, body mass index; PCI, percutaneous coronary intervention; MI, myocardial infarction.

**Supplementary Table S3**. Sensitivity analyses

| Sensitivity analyses | N | Β (95% CI) | P value |
| --- | --- | --- | --- |
| Multiple imputation | 1000 | 0.132 (0.058, 0.206) | <0.001 |
| Excluding prior PCI | 567 | 0.107 (0.017, 0.198) | 0.020 |
| Excluding extreme CHG values beyond the mean±3 SD | 750 | 0.121 (0.024, 0.217) | 0.014 |
| Excluding acute MI during the index hospitalization | 676 | 0.094 (0.003, 0.185) | 0.045 |

β coefficients represent the change in log-transformed (Gensini score + 1) per 1-SD increase in the CHG index. CHG index, cholesterol-high-density lipoprotein-glucose index; CI, confidence interval; PCI, percutaneous coronary intervention; MI, myocardial infarction; SD, standard deviation.

**Supplementary Figure S2.** ROC curves of progressively adjusted models for identifying high coronary atherosclerotic burden

ROC, receiver operating characteristic; AUC, area under the curve; CHG index, cholesterol-high-density lipoprotein-glucose index; FBG, fasting blood glucose; TC, total cholesterol; HDL-C, high-density lipoprotein cholesterol; TyG index, triglyceride-glucose index; AIP, atherogenic index of plasma.

**Supplementary Table S4.** Bootstrap-corrected discrimination and calibration performance

| Model | Bootstrap-corrected AUC | Corrected Brier score | Corrected calibration intercept | Corrected calibration slope |
| --- | --- | --- | --- | --- |
| basic model | 0.594 (0.556, 0.631) | 0.2201 | 0.005 (-0.151, 0.176) | 0.638 (0.467, 0.822) |
| basic model + CHG index | 0.612 (0.574, 0.649) | 0.2169 | 0.005 (-0.152, 0.181) | 0.676 (0.509, 0.865) |
| basic model + FBG | 0.607 (0.568, 0.643) | 0.2179 | 0.005 (-0.149, 0.181) | 0.659 (0.489, 0.841) |
| basic model + TC | 0.593 (0.553, 0.628) | 0.2203 | 0.005 (-0.150, 0.179) | 0.633 (0.466, 0.817) |
| basic model + HDL-C | 0.602 (0.562, 0.639) | 0.2194 | 0.005 (-0.152, 0.183) | 0.646 (0.482, 0.823) |
| basic model + TC/HDL-C ratio | 0.602 (0.564, 0.636) | 0.2189 | 0.006 (-0.152, 0.184) | 0.654 (0.490, 0.834) |
| basic model + TyG index | 0.596 (0.556, 0.633) | 0.2197 | 0.005 (-0.154, 0.182) | 0.642 (0.476, 0.824) |
| basic model + AIP | 0.595 (0.555, 0.632) | 0.2202 | 0.005 (-0.154, 0.183) | 0.635 (0.467, 0.818) |

AUC, area under the curve; CHG index, cholesterol-high-density lipoprotein-glucose index; FBG, fasting blood glucose; TC, total cholesterol; HDL-C, high-density lipoprotein cholesterol; TyG index, triglyceride-glucose index; AIP, atherogenic index of plasma.

**Supplementary Table S5.** Bootstrap-corrected incremental value of CHG index beyond the basic clinical model for identifying high coronary atherosclerotic burden

| Model | Bootstrap-corrected AUC | P value | Continuous NRI | P value | IDI | P value |
| --- | --- | --- | --- | --- | --- | --- |
| basic model | 0.594 (0.556, 0.631) | - | - | - | - | - |
| basic model + CHG index | 0.612 (0.574, 0.649) | 0.068 | 0.129 (-0.023, 0.284) | 0.106 | 0.015 (0.003, 0.021) | 0.022 |

AUC, area under the curve; CHG index, cholesterol-high-density lipoprotein-glucose index; NRI, net reclassification improvement; IDI, integrated discrimination improment.

**Supplementary Figure S3.** DCA of the basic clinical model and the basic clinical model incorporating the CHG index.

(A) Apparent DCA derived from the original study population. (B) Bootstrap-corrected DCA based on 1,000 bootstrap resamples. DCA, decision curve analysis; CHG index, cholesterol-high-density lipoprotein-glucose index.
